# Home Literacy and Numeracy Environments in Asia

**DOI:** 10.3389/fpsyg.2021.578764

**Published:** 2021-03-11

**Authors:** Sum Kwing Cheung, Katrina May Dulay, Xiujie Yang, Fateme Mohseni, Catherine McBride

**Affiliations:** ^1^Department of Early Childhood Education, The Education University of Hong Kong, Hong Kong, China; ^2^Department of Education, University of Oxford, Oxford, United Kingdom; ^3^Faculty of Psychology, Beijing Normal University, Beijing, China; ^4^Department of Psychology, The Chinese University of Hong Kong, Hong Kong, China

**Keywords:** home literacy environment, home numeracy environment, parents, young children, Asia

## Abstract

The home learning environment includes what parents do to stimulate children’s literacy and numeracy skills at home and their overall beliefs and attitudes about children’s learning. The home literacy and numeracy environments are two of the most widely discussed aspects of the home learning environment, and past studies have identified how socioeconomic status and parents’ own abilities and interest in these domains also play a part in shaping children’s learning experiences. However, these studies are mostly from the West, and there has been little focus on the situation of homes in Asia, which captures a large geographical area and a wide diversity of social, ethnic, and linguistic groups. Therefore, this paper aims to review extant studies on the home literacy and numeracy environments that have been conducted in different parts of Asia, such as China, the Philippines, India, Iran, Turkey, and the United Arab Emirates. Specifically, we explore how parents in these places perceive their roles in children’s early literacy and numeracy development, the methods they regard as effective for promoting young children’s literacy and numeracy learning, and the frequency with which they engage their young children in different types of home literacy and numeracy activities. We also examine studies on the relationship of the home literacy and numeracy environment with young children’s developmental outcomes, and the effectiveness of parent training programs to improve the home literacy and numeracy environments in these contexts. By examining potential trends in findings obtained in different geographical areas, we can initially determine whether there are characteristics that are potentially unique to contexts in Asia. We propose future research directions that acknowledge the role of cultural values and social factors in shaping the home learning environment, and, by extension, in facilitating children’s early literacy and numeracy development.

## Introduction

The home learning environment encompasses the beliefs and attitudes that parents hold about children’s learning, as well as their practices for helping children to learn at home (Dearing and Tang, 2009). Over the past several decades, considerable attention has been given to the learning environments that support children’s reading and mathematics skills – also known as the home literacy environment and the home numeracy environment, respectively (e.g., Manolitsis et al., 2013; Niklas and Schneider, 2013, 2014; Skwarchuk et al., 2014; Napoli and Purpura, 2018). There is wide support for the idea that they play an imperative role in early literacy and numeracy development, which, in turn, can influence academic attainment in subsequent school years (Melhuish et al., 2008; Manolitsis et al., 2013; Bywater et al., 2015; Niklas and Schneider, 2017).

To date, the majority of studies and frameworks that inform our understanding of home literacy and numeracy environments have been conducted in Western societies; relatively little attention has been given to the situation in Asia. This matters because from theoretical perspectives, culture plays a critical role in the home environments and child development. According to social constructivism theory, knowledge is situated in specific sociocultural contexts, and children construct knowledge through communications and interactions with more experienced members of the society (Vygotsky, 1978; Simina, 2013). Moreover, the ecological systems theory posits that larger social contexts (e.g., communities and education policies) and cultural values can affect children by shaping their immediate environments, such as the ways their parents interact with them and the physical environment that their parents provide to them (Bronfenbrenner, 1979). The cultural practices approach to development further suggests that people and contexts co-create each other (Miller and Goodnow, 1995). Whereas the cultural practices of a specific place shape its citizens, cultural practices may be reproduced or transformed upon agreement within a particular group, and this mechanism can operate at societal, community, and family levels (Miller and Goodnow, 1995). Literacy (which can broadly include reading, mathematical, as well as scientific literacies) is one kind of cultural practices, and it can be conceptualized as the skills necessary for a specific place at a particular time point to meet certain purposes (Street, 1993; Kell and Kell, 2014). In any place, (a) certain kind(s) of literacy (e.g., a specific language) may be more privileged over others, and it can maintain its dominant position through the government policies, school curricula, and/or pedagogies advocated (Kell and Kell, 2014). In light of the above, joint parent-child literacy and numeracy activities have the potential to contribute to children’s acquisition of literacy and numeracy skills; however, the content of the activities and the knowledge derived from the activities may vary across families, communities, and the wider sociocultural contexts.

Asia is the largest continent by geographical area and by population on Earth (United Nations, 2019). According to the United Nations geoscheme, it can be divided into five sub-regions, namely Eastern Asia (e.g., China, Japan, and Korea), Southeastern Asia (e.g., Philippines, Thailand, and Vietnam), Southern Asia (e.g., Bangladesh, India, and Nepal), Central Asia (e.g., Kazakhstan, Kyrgyzstan, and Tajikistan), and Western Asia (e.g., Israel, Turkey, and United Arab Emirates; United Nations, 2020). Whereas a number of economies, particularly clustered within Eastern and Western Asia, are classified as high-income economies, a larger number of economies in Asia are within the low- and middle-income classification (World Bank, 2019). Such variations in income levels should be taken into account when understanding the home learning environments in Asia, because the economic strength of a place can have implications for the educational policies and child-bearing practices there. At the macro level, compared to low- and middle-income economies, high-income economies may place a greater demand on educational attainment on its workforce and are able to invest more resources in education (Cheng, 2001). At the micro level, children in low- and middle-income economies are more prone to school dropout than their peers in high-income economies, because their families may not be able to afford the costs for schooling, and teens may be engaged in income-earning activities instead (Cheng, 2001; Joshi, 2015). On the linguistic landscape, there are great variations in the languages spoken and scripts used across Asia (Adamson, 2018). In numerous places, children are supported in learning two or more languages from the early grades (Joshi, 2015; Adamson, 2018; Wang, 2018). For example, in Singapore, English has been adopted as the major language of instruction in schools, so as to prevent the dominant ethnic Chinese there from being privileged and ensure opportunities (including educational and social ones) for all children; and each child is taught their mother tongue as a second language (Kell and Kell, 2014). Broadly speaking, most Asian societies are collectivistic in nature: Support among extended family members is common, and members within the society feel some responsibility for one another (Hofstede, 2001; Hofstede et al., 2010). Specifically, in Eastern Asia, as well as Vietnam and Singapore in Southeastern Asia, under the influence of Confucianism, formal education and success in examinations are typically regarded as the major ways to move up the social ladder and are, thus, heavily emphasized (Cheng, 2001). There is keen academic competition, and parents are generally very willing to invest in children’s education, including shadow education (i.e., extracurricular classes for improving children’s academic performance; Yamamoto and Brinton, 2010; Byun et al., 2012; Bray, 2013). In line with these observations, children in some of these places (e.g., Hong Kong, Macao, Korea, Japan, and Singapore) tend to excel in international assessment studies such as the Programme for International Student Assessment (PISA), Progress in International Reading Literacy Study (PIRLS), and Trends in International Mathematics and Science Study (TIMSS; Mullis et al., 2016, 2017; Organisation for Economic Co-operation and Development, 2020). These assessment results, however, have to be interpreted with caution, because the “local” language used for test administration is determined by the gatekeepers, and such “privileged” language may not be the first language of all children living in a place (Kell and Kell, 2014). Despite the above, cultural diversities can exist across and within territories in each sub-region (Miller and Goodnow, 1995). In view of the complex sociocultural contexts of Asia, it is worthwhile to look more closely into the home learning environments there.

What are the characteristics of home literacy and numeracy environments in Asia? How are children’s literacy and numeracy skills supported in different places there? With these questions in mind, this review paper has four major goals: (1) to uncover the learning-related beliefs and attitudes held by parents in Asia; (2) to present a portrait of the home literacy and numeracy practices in Asia; (3) to examine relations between home literacy and numeracy environments and children’s learning in Asia; and (4) to explore the effectiveness of family-based interventions to improve home literacy and numeracy environments in Asia. Here, it should be noted that our primary goal is not to highlight and explain how the home literacy and numeracy environments in Asia are distinct from those in the West, because there have only been minimal studies making direct in-depth comparisons between the home learning experiences of children with comparable demographic backgrounds in Asia and the West, making it insufficient to draw conclusions regarding the exact East-West differences, if any, and the underlying cultural mechanisms. Instead, by addressing the above four issues, we seek to further our understanding and appreciation for the wide diversity in the ways in which homes facilitate children’s literacy and numeracy learning across sociocultural contexts in Asia.

To achieve our research goals, in the following, we review studies published in English that examine the home literacy and numeracy environments experienced by children aged eight or below in Asia (as defined by the United Nations geoscheme). However, results of large-scale international datasets of older children may be reported to provide additional context for cross-context comparisons. Studies that were conducted with Asian parents and children living in places other than Asia (e.g., migrant families in Western societies) were beyond the scope of this article and, thus, are not discussed here. To obtain a picture of the more recent situation in Asia, only studies published in 2001 and thereafter (i.e., the most recent two decades) were included in this review. Where possible, we cite studies that involve children living in different sub-regions of Asia. Study searches were conducted using APA PsycINFO, ERIC, PubMed, and Google Scholar. Examples of keywords used for our search include home learning/educational environment, home language/literacy environment, home math/numeracy environment, home learning/educational activities, home language/literacy activities, home numeracy/math activities, home learning/educational practices, home learning/educational resources, home language/literacy resources, home numeracy/math resources, educational toys, parental learning beliefs/attitudes, parent-child (joint) activities/play, parent-child interactions, math talk, parental/family involvement, parent training/coaching, and parent intervention. This procedure yielded the majority of studies included in this review. Given that there is a lack of studies for certain places (especially those in Southern, Southeastern, Central, and Western Asia) based on the search strategy stated above, we also considered gray literature (e.g., student dissertations, reports by non-governmental organizations such as Save the Children, UNICEF, and RTI International) about those places in our review; these reports were found either using Google with the same set of search keywords or through hand searches of the websites of the aforementioned organizations. The following sections are organized around our four research goals.

## What Learning-Related Beliefs and Attitudes Do Parents in Asia Hold?

In this section, we explore various learning-related beliefs and attitudes held by parents in Asia. These include parents’ perceptions of the nature and importance of academic achievement, their perceptions of the importance of parental involvement in children’s learning, their expectations of children’s academic abilities, and their beliefs about effective methods for teaching and learning.

### How Important Is Academic Achievement for Parents in Asia?

Consistent with Confucian values, Chinese parents have traditionally considered it important for children to excel in their studies (Luo et al., 2013). Parents place a high value on diligence, academic training, concentration, and persistence in the learning process, as these attributes are perceived as keys for academic success (Li, 2012). Consistent with these values, cross-cultural studies have demonstrated that parents in Taiwan and Hong Kong put greater emphasis on young children’s academic attainment than do parents in the United States and United Kingdom (Pearson and Rao, 2003; Wang and Tamis-Lemonda, 2003). Similarly, in one study, parents in Mainland China expressed high aspirations for their children’s academic achievement, with about 76.38% of parents of 3–6-year-old children in rural areas and about 86.05% of those in urban areas expecting their children to get a bachelor’s degree (Su et al., 2020). In another study in Japan, all of the mothers interviewed hoped that their preschool children would like going to school, have high motivation to study, and excel in academic performance (Yamamoto, 2015).

In the case of Mainland China, parents’ emphasis on children’s academic success can also be attributed to broad societal factors; these include the one-child policy and keen competition following the transition from a planned to a free market economy (Luo et al., 2013). Similarly, socioeconomic motivations have been observed in samples from South and Southeastern Asia. Among low-income families in India, Vietnam, and the Philippines, parents aspire for children to finish school to escape poverty (Boyden, 2013) or to provide educational opportunities that were not available to the parents’ or grandparents’ generations (Tatel-Suatengco and Florida, 2018). However, inequalities related to gender in India and minority ethnic status in Vietnam are examples of additional hurdles for children to access educational opportunities in these contexts (Boyden, 2013; Rao et al., 2013). Parents also make schooling decisions against a backdrop of sociopolitical tension within their respective society. For example, in Western Asia, a sample of upper-middle class Palestinian parents in Israel expressed aspirations for better educational opportunities and multicultural education for their children, thus informing their decision to enroll their children in a Palestinian-Jewish bilingual primary school (Bekerman and Tatar, 2009).

Though our selection of studies highlighted here is rather limited, collectively, they suggest that parents generally strongly desire for their children to obtain a good education. However, cultural values, socioeconomic motivations, and sociopolitical factors can create a wide variety of situations among families in Asia that influence the educational decisions they make for their children.

### Should Parents Be Involved in Children’s Learning?

While Asian parents generally have high aspirations for their children’s academic achievement, to what extent do they believe that they play a direct role in their children’s learning? In Korea, parents generally subscribe to the belief that helping their children to learn is one of their major responsibilities (Lee, 2002; Park and Kwon, 2009). Parents in Hong Kong and Mainland China tend to endorse the idea that parents should engage their preschool children in learning activities (such as language and cognitive activities) at home, so as to enrich their knowledge base and promote their all-round development (Lau et al., 2012). In Iraq, one study showed that most parents of 4–7-year-old children strongly agreed that it was essential to teach literacy skills to their children at home (Okello and Mahammed, 2019). Parents in Oman were also conscious that children’s educational problems could be solved gradually over time through the cooperation among children, families, and schools (Tekin, 2015).

However, parents in some studies appear to view their responsibilities as relatively small in their children’s early formal learning. For example, in a study involving six parents in the United Arab Emirates, the parents showed little awareness of the importance of parent-child interactions and stimulating early home environments to support their children’s emergent writing skills (Tibi et al., 2013). The Kazakhstani mother in a case study by Amantay (2017) also expressed the idea that literacy was “something special” (p. 31) and believed that parents had little to do at home to promote children’s literacy development. In focus group discussions, parents in Laos expressed a belief that pre-primary teachers, not families, are responsible for fostering children’s basic literacy and numeracy skills before they enter primary school (Vongxaiya, 2019).

It is not clear whether or not the diverging beliefs reported in these samples reflect broadly-held views within their respective societies. It is also unclear what factors shape these beliefs. Nonetheless, it is important to explore in more depth how parents’ perceptions of their role in their children’s learning are related to the home literacy and numeracy environment they create and to children’s developmental outcomes more generally across countries and territories.

### What Should Children Know Before Entering Formal Education?

Generally speaking, Asian parents place great importance on preparing their children to enter formal education. For example, in Korea, reading and writing are typically regarded by parents as important skills that should be developed even before entering primary school (Lee, 2002). In Hong Kong, “high interest in reading” and “basic writing skills” were rated as the two most important qualities required for a smooth transition from kindergarten to primary school in one study (Chan, 2012). Parents in Laos also view literacy and numeracy competencies as essential components of school readiness (Vongxaiya, 2019). In Mainland China, parents generally acknowledge the importance of learning about “numeracy and quantity” (e.g., counting one to nine objects and telling the amount) and “geometry and space” (e.g., the main characteristics of geometric shapes) before entering school, though skills related to “numeracy and quantity” are expected to be mastered slightly earlier than are skills related to “geometry and space” (Pan et al., 2018).

Beliefs about early childhood education are, of course, influenced strongly by the ideas and policies of one’s government. For example, although parents in a study in Turkey were familiar with the importance of providing developmentally appropriate practices to their preschool children, they gave the lowest rating to children’s emergent literacy development as compared to other items on the scale (Demircan and Tantekin-Erden, 2015). This relatively low rating was attributed to early childhood education guidelines released by the education ministry, which did not identify emergent literacy as a major goal in preschool. Similarly, in a large-scale study in Nepal, mothers generally believed that they should start reading to children at 1–3 years old, which was older than the recommended milestone of 4–6 months in the United States (Shrestha et al., 2017). The overall results of the study were interpreted to reflect a relatively low level of knowledge about child development among Nepalese mothers and were identified as an area for intervention. In Cambodia, most parents believed that preparing children for school involved buying bags and stationery; in contrast, very few mentioned preparing children by building their basic literacy and numeracy skills in one study (Howell et al., 2016). The authors highlighted the need to address this knowledge gap through parent education programs. Thus, parents’ beliefs about what constitutes “school readiness” likely reflects a confluence of cultural values, socioeconomic opportunities, and sociopolitical factors.

### What Methods of Learning Are Considered Effective?

In East Asia, evidence suggests a tension between “traditional methods” and “Western ideas” around child-centered learning and a focus on non-academic domains of child development. In Chinese societies, rote memorization and drill-and-practice are traditionally relied upon for literacy and numeracy learning (Rao et al., 2010; Lam and McBride-Chang, 2013). In a study conducted in Hong Kong, Chan (2016) revealed that some parents still preferred the use of traditional drill-and-practice approach to help kindergarteners learn, despite the active promotion of constructivist learning methods in the city over the past decade. In contrast, parents in Mainland China have demonstrated an increased awareness of the importance of play during early childhood years (Rao and Li, 2009; Lin and Yawkey, 2013). Lin and Li (2019) examined the extent to which mothers of Mainland Chinese children value pre-academic activities and free play, and their results aligned with the idea that parents in Mainland China have diverse views on what should be emphasized in children’s development. In their study, about 44% of the samples was classified as eclectic mothers, who place high value on both pre-academic activities and free play (Lin and Li, 2019). Of the remaining sample, half were classified as traditional mothers (i.e., valuing pre-academic activities highly but free play at a lower level), and half of them were classified as contemporary mothers (i.e., valuing free play highly but pre-academic activities at a lower level).

In traditional Chinese culture, play is not favored, as it is regarded as distracting children from pre-academic learning (Leung, 2011; Luo et al., 2013). With the promotion of the idea of “learning through play” in recent years, parents in Mainland China, Hong Kong, and Taiwan nowadays are more aware of the role of play in children’s learning (Rao and Li, 2009; Fung and Cheng, 2012; Lin, 2013; Lin and Yawkey, 2013). Nonetheless, the study of Fung and Cheng (2012) showed that some Hong Kong parents still have some concerns about the extent to which play-based learning can build school readiness skills among children.

Similarly, Lee and Kim (2016) found that mothers in Korea gave a slightly higher endorsement of the behaviorist approach than the constructivist approach to mathematics learning; this is inconsistent with the child-centered educational practices recently advocated there. Though “raising a child with good socio-emotional competence” was considered by upper-middle-class mothers as one of the most important parenting goals, parents reportedly spend most of the time with their preschool children on teaching them academic skills (Park and Kwon, 2009, p. 58). This discrepancy between parenting beliefs and behaviors, to a certain extent, reflects the fact that, though Korean mothers recognize the importance of children’s socio-emotional development, considerable attention is still paid to children’s academic achievement.

There are fewer studies pertaining to these topics outside of East Asia. However, mothers in Bangladesh (Mehnaz, 2013) and parents in the Philippines (Leer and Teodosio, 2016) appear to agree that play stimulates children’s literacy development. In addition, while most families in India appear to believe that children can learn skills through play (Bora et al., 2018), there was less consensus with this idea as compared to other attitudes measured. Furthermore, Cypriot parents demonstrate inconsistent attitudes toward play and learning. They value play more than academic training; however, the type of play they organize for their children tends to be more academically oriented, rather than play-oriented (Shiakou and Belsky, 2013). Broadly speaking, studies suggest that play-based learning and constructivist approaches as a route for early literacy and numeracy learning are being actively promoted in Asia, whether through initiatives backed by the education system or international non-government organizations; however, its uptake among parents varies considerably across contexts.

## What are the Home Literacy and Numeracy Practices of Parents in Asia?

In the following section, we begin by looking into various aspects of home literacy and numeracy practices of parents in Asia, including the number and types of learning resources they provide for children at home, the frequency with which they engage children in different types of learning activities, the content and style of parent-child interactions during home learning activities, and the family members involved in home learning activities. Following this, we identify factors that influence parents’ home literacy and numeracy practices.

### What Kinds of Literacy and Numeracy Resources Are Available and How Many?

At home, there are a variety of educational resources that parents can provide for children to promote their literacy development (e.g., storybooks, letter flash cards, literacy workbooks, board games, card games, and computer games) and numeracy development (e.g., number charts, counting picture books, number workbooks, and games; e.g., Cheung et al., 2018; Dulay et al., 2018). It is natural to expect that children in high- and upper-middle-income economies tend to have a greater number of home learning resources than those in low- and lower-middle-income economies. For instance, in a study conducted in Korea, households of 4–5-year-old children, on average, reportedly contained, on average, 60–100 children’s books (Kim, 2009). In Hong Kong, all kindergarten children in the study of Chow et al. (2010) had at least 10 Chinese storybooks at home, with 30–49 storybooks available on average. In contrast, in the Philippines, a study showed that children in low- and middle-income families only had 1–9 storybooks on average (Dulay et al., 2019). Although the samples in the aforementioned studies are not directly comparable due to differences in study aims and recruitment methods, these findings are generally consistent with an analysis of the Progress in International Reading Literacy Study 2001 which reported a higher national average of the number of books at home (among fourth graders) in high-income economies (e.g., Singapore) compared to low- and middle-income economies (e.g., Iran; Park, 2008).

A similar trend can be observed within the spectrum of low- and middle-income economies. Studies from economies in the upper middle-income category generally report high rates of the availability of learning resources at home. In a sample in Sri Lanka, for example, more than 90% of third graders reported having storybooks and newspapers at home (Wickramasekara et al., 2014). As demonstrated in two studies, nearly all children in Jordan and 78% of children in Lebanon were even found to own four or more types of reading materials and toys (Queen Rania Foundation, 2017; Save the Children, 2017). In contrast, in low- to lower middle-income economies, such as India, Nepal, and Indonesia, there is evidence that fewer than half of families own print materials, storybooks, and number toys (Bhattacharjea et al., 2011; Research Inputs and Development Action, 2016a,b; Mayasya, 2017; Bora et al., 2018).

The above observations are also consistent with an analysis by the Multiple Indicator Cluster Survey Round 3 (MICS3), which compared the availability of formal learning resources (e.g., children’s books and store-bought toys) in households with children aged 5 or below in 28 developing countries (Bradley and Putnick, 2012). Of the nine Asian countries investigated, Kazakhstan, Uzbekistan, Kyrgyzstan, and Thailand (from highest to lowest scoring) were above the grand mean, whereas Mongolia, Syria, Vietnam, Tajikistan, and Yemen (from highest to lowest scoring) were below the grand mean.

Though it is easy to assume that the home learning environments in low- and lower-middle-income economies are impoverished and devoid of materials for cognitive enrichment, whereas homes in high-income economies are always well-resourced, empirical studies suggest a wider variety of trends across families within a sample, as well as across samples within a country. For example, in a case study involving six mothers of 4-year-olds in the United Arab Emirates, three of the participating mothers often provided children with literacy materials, two of them only sometimes did so, and one of them never did so (Tibi et al., 2013). Samples in Singapore also varied in the reported availability of books, with fewer than 10 children’s books in a sample of mixed ethnicities (Mascarenhas et al., 2017), an average of 10–29 Chinese language books among Singaporean Chinese families (Li et al., 2016), and an average of 10–30 Mandarin books and 30–60 English books among Chinese-English bilinguals (Sun, 2019).

In a qualitative study with five middle-class and upper-middle class mothers of children aged 3–4.5 in Bangladesh, all of them reported having counting, rhyming, and letter books but not illustrated storybooks (Mehnaz, 2013). In contrast, as shown in a survey with 1,856 families with 4-year-old children in Bangladesh, only about 47% had storybooks, 39% had drawing/writing materials, and 23% had number number/counting toys or games (Spier et al., 2018). Among families with low education levels in Iraq, 52% reported having four or more types of toys, as opposed to only 13% for types of reading materials (Okello and Mahammed, 2019). In a low- and middle-income sample from the Philippines, whereas about 20% of families indicated that they have no numeracy-related educational games at home, about 15% of families reported having 10 sets or above of such games (Cheung et al., 2020).

Taken together, the cited studies suggest substantial variation between and within contexts in the number of home learning resources available to children. Furthermore, there appear to be variations in the specific types of literacy or numeracy materials that families own, perhaps in part reflecting what families consider essential to their children’s learning.

### What Literacy and Numeracy Activities Are Carried Out at Home and How Often?

At home, parents can involve children in various learning activities. Some of them involve direct and intentional teaching of literacy and numeracy skills and can be termed as formal (or direct) learning activities (Skwarchuk et al., 2014). Examples include helping children to read words, introducing new words and their definitions, writing numbers, and practicing simple sums (Skwarchuk et al., 2014). Some others are called informal (or indirect) learning activities, because teaching literacy and numeracy skills is not the major goal of such shared activities but may emerge incidentally (Skwarchuk et al., 2014). Examples include reading books, telling stories, and playing number board and card games (Mullis et al., 2016).

In contrast to the trends observed in the number of home resources available, large-scale surveys have revealed that parents of high- and upper-middle-income economies do not necessarily engage children in early literacy and numeracy activities more frequently than those in low- and lower-middle-income economies. In the TIMSS 2015 (Mullis et al., 2016), parents of fourth graders were asked to report their frequency of engaging children in 16 formal and informal learning activities (e.g., reading aloud signs and labels, playing word games, and playing with number toys) at home before their children entered primary school. Results showed that parents in Kazakhstan and Korea often did so, whereas parents in Bahrain, United Arab Emirates, Qatar, Singapore, Jordan, Saudi Arabia, Kuwait, Indonesia, Iran, Turkey, Oman, Chinese Taipei, Japan, and Hong Kong only sometimes did so (Mullis et al., 2016). Using MICS3 data, Zainiddinov and Habibov (2019) compared mothers’ average interaction time with their children under 5 years old (e.g., time spent on reading books, telling stories, and practicing naming and counting) in various countries in Central Asia. The greatest amount of mother-child interaction time was found in Turkmenistan and Uzbekistan, followed by Tajikistan, Kazakhstan, and Kyrgyzstan (Zainiddinov and Habibov, 2019).

Among the various home literacy activities, some seem to be more popular than others. In a cross-cultural comparison between home environments in Iran and Germany, Iranian children were found to engage more frequently in learning poems, rhymes, and songs, but less frequently in book reading, than did German children (Aminipour et al., 2018). In a low- and middle-income sample in Korea, parents, on average, helped children with homework about three to four times a week, taught children Korean alphabet letters/symbols (Hangul) and literacy and read books with children about once a week, but brought children to the library or bookstore only about once a month (Kim, 2009). In Japan, a study showed that parents taught first graders character/kanji names, word reading and character writing a few times a month, and read to children about 5–30 min per day on average (Inoue et al., 2018). In some places, families tend to prioritize the direct instruction of literacy skills over storybook reading and storytelling, as is the case in samples from Cambodia (Howell et al., 2016) and Indonesia (Mayasya, 2017). In contrast, trends have varied across samples in Nepal, with one study reporting more letter teaching than storybook reading (LeVine et al., 2012), a second study reporting that a majority of parents engage in storytelling, book reading, and teaching letters (Research Inputs and Development Action, 2016a), and a third study reporting higher rates of reading and storytelling over the teaching of letters (Research Inputs and Development Action, 2016b). In Bangladesh, two studies have shown that a majority of parents provide direct teaching of letters at home (Pisani et al., 2017b; Spier et al., 2018). However, reports of oral storytelling and book reading slightly diverged in the two studies: 41–55% of mothers in the study by Pisani et al. (2017b) reported engaging in these activities; 68–69% of parents reported doing so in the study by Spier et al. (2018).

Furthermore, parents may rely more on formal than informal activities to facilitate children’s second language acquisition at home, at least in Hong Kong. In a study conducted with Hong Kong kindergarteners and their parents, instructing children to do English homework was the activity that most frequently occurred, followed by teaching the recognition and writing of English words, watching English educational CD-ROMs, and shared reading (Yeung and King, 2016). Likewise, in another study conducted with Hong Kong parents of 5–8-year-old children, about 72% of them reported engaging in some English learning practices with children at home (Forey et al., 2015). Among the six activities under investigation, teaching English word reading was more prevalent than watching English programs, reading English stories, conversing in English, singing English songs, or playing English games (from highest to lowest frequency; Forey et al., 2015).

What types of home numeracy activities are children more likely to participate in? Few studies have focused on this topic in samples from Asia; however, there may be variations in the formal and informal numeracy activities practiced in different places. In Hong Kong, number skills activities (e.g., printing numbers, counting objects, and learning simple sums) tend to occur more frequently than number book activities, mathematical games (e.g., playing board games with dice or spinner), and application activities (e.g., using calendars and dates; e.g., Huang et al., 2017). In contrast, Cheung et al. (2020) found that in the Philippines, the three most common home numeracy activities mentioned by parents were talking about money and the prices of goods, teaching how to do math in one’s head, and talking about counting and practicing counting skills during everyday activities, whereas the three least common home numeracy activities were playing number card games, board games, and/or computer games, completing exercise books related to numbers, and talking about the meaning of numbers during everyday activities.

Finally, are parents more likely to engage children in literacy or numeracy activities at home? Numeracy learning may be especially important for Indian families, with 98% of caregivers reporting that they have taught their children numbers, in contrast to 45–61% for teaching letters (Bora et al., 2018). The reverse pattern, however, was observed in the Philippines when we compared the findings obtained from the studies of Cheung et al. (2020) and Dulay et al. (2019): Home literacy activities, on average, were reported to occur more frequently than home numeracy activities among Filipino families. The overall picture that emerges is of considerable heterogeneity in the frequency and variety of home literacy and numeracy practices in Asia, with no clear trends across and within contexts.

### How Do Parents and Children Interact During Literacy and Numeracy Activities?

Comparatively, studies that focus on the content and style of parent-child interactions during home learning activities are scarce and scattered. In Israel, Korat et al. (2012) found that when Arabic-speaking mothers engaged kindergarten children in book reading at home, “paraphrasing the text” and “discussing the story” were the two most common maternal mediation behaviors, whereas “talking about illustrations,” “telling the story in spoken language,” and “discussing about the written system” rarely occurred. This probably happens at least in part because of the diglossic nature of Arabic. Arabic-speaking children usually do not understand much about the formal written language when they are young; thus, mothers are inclined to spend much time on helping children to understand the meaning of the story, leaving little room for in-depth discussions on things beyond the text (Korat et al., 2012). On the topic of children’s early writing, the strategies that parents employ to help their children to write partly depend on their writing system. Observations of parent-child dyads in Israel have revealed that mothers utilize different strategies to help their child to write in Hebrew, ranging from writing the word for the child to helping the child to break down the component sounds of words and connect each with the appropriate letter (Aram and Levin, 2001). In another study, asking children to write letters was found to be the most frequently used strategy by Israeli mothers when teaching children to write in Arabic; these mothers seldom guided their children to make connections between sounds and letters (Korat et al., 2014). In contrast, a study that focused on Hong Kong mothers identified a different set of scaffolding strategies, given that Chinese characters have a different level of visual complexity and can contain cues to both pronunciation and meaning (Lin et al., 2011). This study demonstrated that stroke-focused strategies (e.g., telling children where a line should go) and component segmentation strategies (e.g., telling children what components a Chinese character is made up of) were the most frequently used strategies (Lin et al., 2011). On the other hand, visualization strategies and strategies that focused on shared phonetic components were the least frequently used (Lin et al., 2011). One potential reason for these finding is that the drill-and-practice method is traditionally adopted to teach children how to write in Chinese societies (Wu et al., 1999). Despite the fact that the scripts involved in the above studies are different, the findings seem to provide converging evidence that during joint writing, not all parents carry out higher levels of mediation that helps children to understand the writing system (i.e., guiding children through the grapho-phonemic encoding process for an alphabetic writing system, drawing children’s attention to the morphological information conveyed by a character for a logographic writing system; Aram and Levin, 2001; Lin et al., 2011; Korat et al., 2014).

On the topic of early numeracy, Cheung and McBride (2017) observed how parents in Hong Kong interacted with their kindergarteners when playing a number board game: Parents varied greatly in their sensitivity to incorporating developmentally and educationally appropriate numeracy elements in their discourse with children (Cheung and McBride, 2017). Specifically, many parents focused on asking children to count aloud the number of moves (which ranged from one to six only), but overlooked the possibility that they could ask children to announce the numbers shown on the board or the numerical distance from one place to another on the board (Cheung and McBride, 2017). In Israel, Tzuriel and Mandel (2020) examined the mathematical discourse between parents and children during joint tasks related to mathematics. Their results showed that “using mathematical language” was the most prevalent, followed by “extending learning with varied mathematical information” and “illustrating the problem and/or solution with visualization strategies.” Detailed observations of such parent-child interactions can provide a valuable window for strategies that parents in Asia may adopt while conducting literacy and numeracy activities with their children and remains an open area for investigation.

### Which Family Members Are Involved in Home Literacy and Numeracy Activities?

Unsurprisingly, mothers in Asia tend to be more likely than fathers to report being involved with their children in home learning activities. For example, in Cambodia, Mongolia, and Timor-Leste, mothers were more likely to report engaging in learning activities at home (including literacy, numeracy, and socioemotional activities) than fathers were (Sun et al., 2018a). In Turkey, Şad and Gürbüztürk (2013) also found that mothers reported providing more support to children for their homework than fathers did. In Hong Kong, mothers report a higher frequency of engaging 5-year-olds in literacy and numeracy activities than fathers did at home (Huang et al., 2017; Xiao et al., 2020).

However, the differences between maternal and paternal engagement might be less clear for home numeracy activities in Hong Kong. In one of the aforementioned studies on 5-year-olds, for example, no significant differences were found between mothers and fathers in their frequency of engaging in number game activities (Huang et al., 2017). In a sample with young children (approximately 3-year-olds), mothers and fathers also reported a similar frequency of engaging children in number skill activities, number book activities, and application activities and fathers reported a higher level of engagement in number game activities (Liu et al., 2019).

Interestingly, numerous studies in Asia have highlighted the role of non-parental family members in promoting home learning activities; namely, siblings and grandparents. In Myanmar, non-parental family members were the most likely to read books or play with the child in one study, for example (Rao et al., 2017). In Mainland China, grandparents’ involvement in the daily care of young children is a tradition, and a recent study showed that between 30 and 40% of 3–6-year-old children in rural and urban areas were taken care by their grandparents in the daytime (Su et al., 2020). Grandparents in Korea read books with young children (Chung and Koo, 2001), whereas grandparents in the Philippines provide children with exposure to the mother tongues (Tatel-Suatengco and Florida, 2018). Siblings have been reported to help with homework in Laos (Vongxaiya, 2019) or to provide help with reading at home in Korea (Chung and Koo, 2001) and in the Philippines (Education Development Center, Inc., 2015). The intergenerational nature of the home literacy environment has also been emphasized in Singapore (Ren and Hu, 2013a,b). These studies demonstrate that the responsibility of creating a home learning environment for children tends to be distributed across different household members in Asia. In some places, such as in Hong Kong, Singapore, and the Arabian Peninsula, foreign domestic workers might even be expected to participate in this role, particularly vis-à-vis English language learning (e.g., Dulay et al., 2017).

### What Drives Parents to Engage in Home Literacy and Numeracy Practices?

Parents can either encourage or hinder children in learning *via* home learning practices. For example, parents’ perceptions of the self and aspirations for their children can play a role in the home environment that they foster. For instance, in Japan, mothers who regarded themselves as bearing responsibility for children’s learning were more likely to engage children in home cognitive and intellectual activities in two studies (Yamamoto, 2016; Yamamoto et al., 2016). Mothers’ occupational aspirations for children have been found to be positively correlated with the amount of cognitive stimulation they give children at home (e.g., reading and playing card and board games with them) in Japan (Holloway et al., 2008), and the frequency with which they check and monitor children’s homework (e.g., spelling, writing, and mathematics practice) in Korea (Kim et al., 2011). Qualitative studies in South and Southeastern Asia have also identified pragmatic and strategic reasons for teaching their children literacy and numeracy skills at home. In Bangladesh, middle and upper-class mothers who were interviewed tend to view the perceived toughness of school admissions processes as a factor that determines how much children should be taught about basic literacy and numeracy skills at home (Mehnaz, 2013). Similarly, the fear of losing in a competitive society is thought to motivate some Chinese families in Singapore to provide a strong learning environment for their children (Ren and Hu, 2013a). In Indonesia, some parents consider English language learning as a key for their children to eventually study overseas (Dharmaputra, 2019). In Singapore, a small sample of Chinese families have shared that they value their cultural identity and language and seek to preserve it at home; nevertheless, most parents additionally value English-language opportunities given the educational system in Singapore (Ren and Hu, 2013a,b).

Parents’ home literacy and numeracy practices are also related to their access to tangible and non-tangible resources. In one study from Mainland China, the higher the family’s socioeconomic status, the greater the number of home literacy resources owned by kindergarteners (Liu et al., 2018). Similarly, parents’ education and occupational status have been linked to preschool children’s readiness through the frequency of parents’ engagement in home learning activities, as well as children’s participation in extracurricular activities; similarly, parents’ income has been linked with preschool children’s school readiness *via* children’s participation in extracurricular activities (Liu et al., 2020; Ren et al., 2020). In Japan, mothers from higher socioeconomic backgrounds have reported a higher frequency of reading to preschool children at home (Yamamoto et al., 2006). In one study from Turkey, parents’ education level and the household income tend to be positively associated with the number of home literacy experiences they provide to their preschool children (Altun, 2019). Consistent with this, another study has found that mothers with higher levels of education are more likely to teach their 3–7-year-old children reading at home than mothers with lower levels of education (Iflazoglu Sabah et al., 2018). In Kazakhstan, Kyrgyzstan, Tajikistan, and Uzbekistan, a large-scale dataset has revealed that the wealthier the families, the greater the number of mother-child interactions (Zainiddinov and Habibov, 2019). In studies from Israel, 5–6-year-olds from low socioeconomic families are found to have fewer educational games related to reading and arithmetic at home than their peers from higher socioeconomic families (Korat et al., 2007). Moreover, mothers from higher socioeconomic backgrounds are also found to discuss more with children about the written system during joint book reading (Korat et al., 2012), and intrude less frequently into the children’s space during joint writing (Aram et al., 2013b).

Finally, parents’ own ability and interest in literacy and numeracy may drive variations in the home environments they provide. In two studies from the Philippines, parents’ own reading and calculation skills, as well as their own interest in literacy and numeracy activities, were each positively correlated with home resources and activities related to the two domains (Dulay et al., 2019; Cheung et al., 2020). On the other hand, when Hong Kong parents were asked about the reasons for not supporting children’s English learning at home, “lack of time” was the most commonly cited, followed by “lack of English teaching skills” and “not knowing English” in one study (Forey et al., 2015).

Overall, studies suggest that parents in Asia engage in home learning practices to cope with the expectations and demands of the societies they live in, and that this can be made easier if they possess adequate economic and social capital as well as skills and interest in the two learning domains.

## Are the Home Literacy and Numeracy Environments in Asia Conducive To Young Children’s Learning?

Presumably, the home learning environment plays a vital role in children’s early literacy and numeracy development. The real-life situation, however, is much more complicated than assumed. In the following, we first examine the extent to which various aspects of the home learning environment in Asia (including the quantity and quality of home literacy and numeracy practices, as well as parents’ beliefs, attitudes, own academic abilities, and interest) are predictive of young children’s learning. Next, we discuss what factors may affect the relation between the home learning environment and children’s development.

### Do Home Literacy and Numeracy Resources and Activities Matter?

#### Home Literacy and Numeracy Resources

Across different places in Asia, there have been studies demonstrating positive correlations between the number of home educational resources available and children’s literacy and numeracy outcomes. Such a pattern of results is evident among studies conducted with children of different ages. Among preschool children, the number of books and reading materials at home has a significant relationship with children’s literacy skills in Japan (Hamano and Uchida, 2012) and early literacy and numeracy scores among preschool children in Thailand (Morales et al., 2016). More broadly, owning other types of literacy materials or child-friendly materials was positively correlated with children’s early literacy and numeracy scores in Vietnam in one study (Pava, 2016) and with children’s vocabulary scores in the Philippines in another (Dulay et al., 2018). Among primary school-aged children, having books at home was related to higher levels of letter knowledge in Bangladesh (Dowd et al., 2017), word reading in Hong Kong (Lau and McBride-Chang, 2005), reading fluency and comprehension in the Philippines (Education Development Center, Inc., 2015), reading fluency in Indonesia (Brown, 2013), and reading fluency in the West Bank and Gaza (Weatherholt et al., 2018) across different studies. Conversely, in Iraq, having fewer toys and learning materials at home was negatively associated with 4–7-year-old children’s performance in literacy and numeracy skills (Okello and Mahammed, 2019). The presence of relevant learning resources also appears to be beneficial for second language acquisition. Exposure to English materials has been found to be a significant correlate of English vocabulary skills among kindergarteners in Hong Kong (Yeung and King, 2016) and Singapore (Sun et al., 2018b). Few studies have analyzed numeracy-related materials as a distinct type of home learning resource, as opposed to grouping them together with literacy materials and toys. However, a similar trend could be expected; for example, the number of numeracy resources available at home had a positive link with 5–8-year-old children’s numeracy competence in the Philippines in one study (Cheung et al., 2020).

#### Home Literacy and Numeracy Activities

Among the different home learning environment variables, the relationship between the provision of home learning activities and children’s learning has received the greatest amount of attention among researchers in Asia. While many studies have found positive relationships between the two, there have also been studies that have demonstrated mixed trends across skill domains.

In general, children’s engagement in book reading at home tends to be positively related to their language and literacy outcomes among kindergarten and early primary grade children; specifically for emergent and conventional literacy skills in Korea (Kim, 2009), early literacy scores in Thailand (Yampratoom et al., 2017), reading skills in Iraq (Brombacher et al., 2012), verbal abilities (including vocabulary, syntax, and conversation) in Israel (Aram and Levin, 2002), and reading fluency in the West Bank and Gaza (Weatherholt et al., 2018). In relation to second language acquisition, English book reading has been positively associated with English language and literacy skills in Hong Kong (Yeung and King, 2016) and in India (Kalia, 2007). Another study in India found that kindergarten children who practiced writing at home had higher year-end English reading achievement scores (Sen and Blatchford, 2001). However, in one example of a non-significant relationship between book reading and children’s literacy, the frequency of book reading was not a significant correlate of first graders’ reading and writing skills in Israel (Aram et al., 2013a).

Studies that have included literacy activities other than book reading likewise have shown that they had positive impacts on various literacy outcomes. Home teaching of English at home was a significant correlate of kindergarteners’ letter knowledge in Hong Kong in one study (Yeung and King, 2016), for example. The provision of various literacy activities (e.g., writing) have also been associated with young children’s reading competence and interest in Singapore (Yeo et al., 2014) and narrative skills in Turkey (Işıtan et al., 2018). In Mainland China, early scaffolding of pinyin knowledge has been associated with subsequent literacy skills (McBride-Chang et al., 2012), and joint parent-child literacy activities in general contributed directly to first graders’ reading performance (Shu et al., 2002).

Home numeracy activities, or home learning activities in general, also have positive associations with children’s numeracy outcomes. In the Philippines, the frequency of home numeracy activities was found to be a positive correlate of 5–8-year-old children’s numeracy skills (Cheung et al., 2020). In Bhutan, the number of home learning activities was also positively related to children’s literacy and numeracy scores, regardless of whether they received early childhood care and development services (Pisani et al., 2017a). In Jordan, conducting more learning activities at home was associated with 3–6-year-old children’s emergent literacy and numeracy skills (Queen Rania Foundation, 2017). However, in one study conducted in Mainland China, neither formal nor informal home numeracy environment dimensions were significant correlates of children’s later mathematical performance (Deng et al., 2015). To account for this, the authors speculated that the home learning environment questionnaire had only captured parental teaching and no other aspects of parent-child interactions (Morrison, 2009).

Some researchers have found differential patterns of relations between home learning activities and different aspects of literacy and numeracy development. For example, a cross-national study involving second to third graders revealed that family members’ engagement in reading activities at home was positively related to children’s letter knowledge, fluency, and comprehension in Indonesia and in the Philippines (Dowd et al., 2017). In Bangladesh, reading engagement was only related to letter knowledge and not the other skills.

In the same way, different types of home learning activities could be more beneficial for some skills than in others. In Mainland China, Chen et al. (2010) found that the amount of formal home literacy experiences (e.g., teaching Chinese characters) was positively correlated with first graders’ reading skills, whereas the amount of informal home literacy experiences (e.g., shared book reading) was positively correlated with first graders’ vocabulary knowledge. In Japan, only parent teaching, but not shared reading, was associated with 5–6-year-old children’s early Hiragana (i.e., Japanese syllabary) spelling acquisition (Inomata et al., 2016). Similar findings have been observed in Western societies, wherein formal and informal activities had differential relations with literacy subdomains (Sénéchal et al., 1998; Frijters et al., 2000). A similar trend has been observed in the numeracy domain. In Mainland China, Zhang et al. (2020) found that preschoolers’ frequency of engagement in informal home numeracy activities (including number game and application activities) predicted their formal mathematical skills and their growth, whereas the frequency of engagement in formal home numeracy activities (including number skill and book activities) was not a significant correlate of formal or informal mathematical skills. Consistent with the overall trend in this review, there are many more studies that have focused on the literacy domain as compared to the numeracy domain. For this reason, we are unable to provide much detail across Asian contexts.

Differential relations have additionally been observed according to the family member who engaged in home learning practices, at least in Hong Kong. In a sample of 5-year-old children, fathers’ frequency of literacy teaching activities, but not mother’s frequency, was a significant correlate of children’s word reading skills (Xiao et al., 2020). In contrast, mothers’ involvement in number skill activities was a positive correlate of children’s abilities to solve written arithmetic problems and mathematical story problems, whereas father-child game activities and application activities were predictive of children’s abilities in solving written arithmetic problems (Huang et al., 2017). Using a younger sample of 3-year-old children, Liu et al. (2019), however, found that only fathers’ involvement in number game activities, but not mothers’ involvement in the four types of numeracy activities or fathers’ involvement in the other three types of numeracy activities, made a unique contribution to children’s number skills. It is currently difficult to explain why such results have emerged; however, it is interesting to consider the roles that different family members play in providing cognitive stimulation to children at home, and what characteristics might make them effective teachers in the home learning context.

In a few cases, the frequency of home learning activities and children’s literacy and numeracy outcomes were observed to be negatively correlated. In Hong Kong, although the frequency of home literacy activities was a significant correlate of second graders’ reading comprehension, parents’ involvement in children’s homework (e.g., dictation, reading, and writing tasks) was shown to be negatively correlated (Law, 2008). In the study by Deng et al. (2015) conducted in Mainland China, it was revealed that parents tended to engage more frequently in shared book reading with first graders and second graders who were reported to have poorer reading skills. In Korea, Kim (2009) also found that after taking frequency of reading into consideration, frequent teaching was negatively associated with preschool children’s scores on measures of phonological awareness, vocabulary, word reading, and pseudoword reading. One potential explanation for these results is that parents were responsive to their children’s learning needs; as such, they provided more support when they discovered that their children were weak in certain skill areas (Kim, 2009).

In the study by Cheung et al. (2020), both home numeracy resources and activities were significant correlates of 5–8-year-old children’s numeracy competence. However, home learning resources and activities did not always have an equally positive association with children’s outcomes. For instance, in the study by Dowd et al. (2017), both reading materials and activities were positively related to children’s letter knowledge in Bangladesh; in contrast, only reading activities were related to the same skill among children in Indonesia and the Philippines. In Mainland China, the number of formal literacy activities was a significant predictor of kindergarten children’s phonological awareness, whereas the number of home literacy resources was a significant predictor of their vocabulary knowledge (Liu et al., 2018). Another study demonstrated not only differential patterns of relations for literacy resources vs. activities, but also between types of home literacy activities. Zhang et al. (2019) found that among 3rd-year kindergarteners in Mainland China, formal literacy experiences were positively linked with reading comprehension *via* pinyin knowledge, but informal literacy experiences were not a significant correlate of emergent literacy skills and reading outcomes. In contrast, exposure to literacy resources was positively linked to reading comprehension through rapid naming, phonological awareness, and vocabulary (Zhang et al., 2019).

#### So Do Home Resources and Activities Matter?

In general, it is reasonable to conclude that both home resources and activities matter for children’s literacy and numeracy development in Asia. It is important for families to own materials that can be used to support children’s learning. As far as home learning practices are concerned, their effectiveness could in part be determined by the appropriateness of the activity to the skill domain that is being targeted, the skill of the family member who is conducting the activities, and family members’ sensitivity to the child’s learning needs.

### Does the Quality of Parent-Child Interactions Matter?

Relatively few studies have examined how the process characteristics of home learning activities are related to young children’s learning outcomes. Findings of the existing studies, however, show that the content and style of parent-child interaction play a critical role in children’s learning outcomes. In a study on shared reading activities in Hong Kong, Lau and McBride-Chang (2005) found that asking questions related to the content of the story during parent-child reading was a significant predictor of second graders’ Chinese character recognition skills. A study in Israel found that after controlling for family socioeconomic status and home literacy environment, mothers’ intrusion into children’s space during joint writing was negatively correlated with children’s alphabetic knowledge, concepts about print, phonological awareness, and vocabulary knowledge (Aram et al., 2013a). In another study, the higher the quality of Israeli mothers’ writing mediation, the better first graders’ early reading and writing skills (Aram et al., 2013a). Similarly, in Hong Kong, mothers’ use of higher-level writing mediation strategies was associated with children’s stronger reading and writing skills (Lin et al., 2011). Supportive parent-child interactions during numeracy activities have also been found to benefit children’s learning behaviors. In Mainland China, Huang et al. (2020) found that mothers’ emotional support was positively correlated with preschoolers’ initiative-taking behaviors (e.g., finding solutions independently and showing persistence when difficulties arise) during math-related application activities. In contrast, father’s cognitive and autonomy support were generally related to children’s initiative-taking behaviors across different types of math-related learning activities (e.g., worksheets, games, and application; Huang et al., 2020). Identifying strategies that are best suited to children across different contexts in Asia, and strategies that parents can confidently and effectively utilize in the home context, will, no doubt, be interesting to researchers, practitioners, and organizations involved in developing intervention programs for and in Asia.

### Do Parents’ Beliefs and Attitudes About Learning Matter?

Several studies have shown that parents’ beliefs and attitudes toward learning can have direct as well as indirect influences on children’s literacy and numeracy outcomes. For example, a study in Mainland China demonstrated that parents’ expectations had indirect positive links with kindergarten children’s word reading skills *via* the amount of formal literacy experiences they provided to children and the number of literacy resources available at home (Liu et al., 2018). Both direct and indirect relationships were evident in studies focusing on the numeracy domain. In Korea, mothers’ attitudes toward math (including enjoyment, anxiety, and self-concept) had indirect links with 4–6-year-old children’s abilities and attitudes through their perceptions of children as active math learners (Lee and Kim, 2016). In contrast, mothers’ constructivist views about mathematical learning were positively correlated with 4–6-year-old children’s abilities and attitudes (Lee and Kim, 2016). In the Philippines, parents’ attitudes toward numeracy, including their beliefs about their teaching abilities and the role of parents and play in children’s learning, were positively associated with children’s interest in numeracy activities (Cheung et al., 2018).

However, one study in Singapore demonstrated a mix of positive and negative relations with children’s reading outcomes. In a study by Yeo et al. (2014), parents’ perceptions of their role in preparing children for formal schooling were associated with kindergarten children’s reading competence and parents’ positive affect shown while parent-child reading was found to be associated with children’s reading interest (Yeo et al., 2014). In contrast, parents’ beliefs about children’s verbal participation during reading were negatively related to these children’s reading competence (Yeo et al., 2014). From the few studies available, parents’ attitudes and beliefs toward literacy, numeracy, and child development are likely to influence the way they behave in terms of fostering their children’s skills at home. Understanding these underlying attitudes and beliefs might help explain the wide heterogeneity of trends observed across and within contexts and might be an interesting avenue for future research.

### Do Parents’ Own Literacy and Numeracy Abilities, Interest, and Practices Matter?

Family members can serve as positive role models for literacy and numeracy behaviors that children could emulate. In Thailand, having a father who could read was associated with better numeracy scores than those whose fathers could not in one study (Morales et al., 2016). In India, adult literacy practices were related to children’s vocabulary scores at the end of the year (Vagh, 2009). In Bangladesh, children who reported seeing more than three family members reading at home were more likely to perform better on reading tasks (Islam et al., 2018). In Hong Kong, having an English-speaking foreign domestic helpers benefited 5-year-olds’ English vocabulary performance (Dulay et al., 2017). However, family members could serve as negative role models in some instances. For example, mothers’ foreign language reading anxiety was positively associated with first graders’ foreign language reading anxiety in Hong Kong in one study (Chow et al., 2017).

Children’s and parents’ literacy and numeracy skills have also been found to be related. In Nepal and the Philippines, parents’ own literacy skills were significantly related to their children’s literacy (LeVine et al., 2012; Education Development Center, Inc., 2015; Dulay et al., 2019). Similarly, in the Philippines, parents’ own computation skills and engagement in mathematical activities had direct links with children’s numeracy skills, as well as indirect links *via* the number of home numeracy resources and the frequency of home numeracy activities (Cheung et al., 2020). Overall, these studies have highlighted the intergenerational nature of literacy and numeracy transmission within families and further emphasize the importance of studying what happens within homes in Asia.

### What Factors Affect the Relation Between the Home Learning Environment and Children’s Development?

Why does the home learning environment sometimes fail to predict young children’s learning outcomes in studies from Asia? One possible reason is that the relation between the home learning environment and young children’s learning outcomes is subject to third variables. For example, there is some evidence that home learning environments might function differently between age groups and over time. In a sample of 3-, 4-, and 5-year-olds in the Philippines, no relationships between home literacy environment factors and children’s literacy and numeracy were found, except for home literacy resources and children’s vocabulary in the 5-year-old group (Cheung et al., 2018; Dulay et al., 2018). When the same cohort was between 5 and 8 years old, there were significant relationships between home literacy activities and children’s oral and print skills (Dulay et al., 2019), and both home numeracy activities and resources were significantly related to children’s numeracy performance at the same time point (Cheung et al., 2020).

Speaking the school language at home has also been highlighted as an important facet of academic achievement. A mismatch between the home language and the school language was associated with worse reading and math outcomes in India in one study (Bhattacharjea et al., 2011), whereas speaking Nepali at home was associated with higher overall scores in a child development assessment in Nepal (Research Inputs and Development Action, 2016a). However, the same research group did not find this same home language advantage in a different location in Nepal (Research Inputs and Development Action, 2016b). This is consistent with a recent systematic review that examined the effects of home language-school language among low-to-middle-income countries around the world, noting heterogeneity in the evidence for a “home language advantage” (Nag et al., 2019, p. 91). The broader challenge of multilingualism and becoming proficient in more than one language has also been a topic of concern for some studies in South and Southeastern Asia. Unsurprisingly, studies in Singapore have demonstrated that greater input in the mother tongue (Li et al., 2016; Li and Tan, 2016; Sun, 2019) and English (Sun et al., 2018b) were both related to higher literacy scores in these respective languages. However, another study in Singapore demonstrated that children could achieve a high degree of proficiency in both languages regardless of the degree of relative home language exposure in the mother tongue and in English (Dixon et al., 2012). There might be compensatory mechanisms that make up for the lack of home language exposure in a particular language. For example, a study in India revealed that reading at home mitigated the impact of low English language exposure on children’s English oral and print skills (Kalia and Reese, 2009).

In a more general sense, it might be possible to identify aspects of home environments in Asia that are especially lacking or challenging in certain places. In the same way, there could be compensatory mechanisms within homes and communities that have not yet been identified and accounted for in the research literature. Nevertheless, researchers and organizations have sought to implement interventions to address perceived gaps in the home learning environment of families living in Asia. These initiatives are discussed in the next section.

## Can Family-Based Interventions Improve Home Environments and Children’s Skills in Asia?

What kinds of home literacy and numeracy interventions have been implemented in Asia and are they effective? Based on the studies considered for this review, we identified two types of intervention programs that aimed to improve children’s literacy and numeracy skills. The first type comprises broad parent education programs. Typically, these cover multiple domains of child development such as nutrition, behavior, discipline, and learning in the cognitive, language, and numeracy domains. The second type is characterized by programs that focus more specifically on children’s literacy and/or numeracy skills. The effectiveness of these two types of intervention programs will be evaluated separately, in the sections that follow.

### How Effective Are Broad Parent Education Programs?

Broad education programs can potentially influence both parents’ behaviors and children’s skills; however, results vary across and within samples. In Bhutan, a broad parent education program resulted in positive gains on learning materials, learning activities, and children’s literacy and numeracy skills (Department of Public Health and Save the Children, 2017). However, the variability in program effects was best exemplified by various programs implemented in Bangladesh. In two studies, mothers that received parent education sessions demonstrated better knowledge about child development and were observed to provide more stimulating talk and activities with their children; thus, they demonstrated a positive change in the home learning environment (Aboud, 2007; Aboud et al., 2013). Interestingly, the earlier program only increased boys’, but not girls’, vocabulary scores (Aboud, 2007), but the latter program resulted in significantly higher cognitive and language scores among all children in the intervention group compared to the control group (Aboud et al., 2013). In another case, changes were observed in some aspects of the home environment, but not others. A preschool program that included a parent education and parent-child reading component resulted in an increased percentage of households with learning materials and stronger attitudes about talking to children; however, the frequency of conducting home learning activities did not increase (Guajardo and Nath, 2016). In the least successful program in this set of studies, parent education sessions and messages for mothers and fathers did not result in increased indices of home activities, home resources, or children’s literacy and numeracy skills (Pisani et al., 2017b).

In contrast, promising short-term and long-term effects were reported in interventions conducted in Western Asia. After joining an early childhood care and development and child protection-focused intervention program in Iraq, participating boys and girls in conflict-affected areas generally outperformed the control group in most developmental domains, and their parents likewise demonstrated better literacy skills than parents in the control group (Hamakareem and Okello, 2019). Parents who participated in the program were also observed to provide more reading and play materials to support children’s literacy and motor development than parents in the control group (Hamakareem and Okello, 2019). Furthermore, one program in Turkey demonstrated how a 2-year mother training program for promoting children’s early literacy, early numeracy, and socioemotional skills, as well as mothers’ own empowerment, could result in long-lasting benefits to children’s academic and behavioral outcomes. In the 7-year follow-up of the Turkish Early Enrichment Program (TEEP), Kagitcibasi et al. (2001) revealed that mother training resulted in higher levels of school attainment, vocabulary scores, parental educational expectations, and better behavioral outcomes for the children (Kagitcibasi et al., 2001). While most of the benefits of mother training in particular had largely disappeared in the 19-year follow-up, the children who received some form of early enrichment demonstrated evidence of positive educational, occupational, and social outcomes later in life (Kagitcibasi et al., 2009).

### How Effective Are Domain-Specific Parent Education Programs?

The second program type appeared to more reliably benefit children’s literacy and numeracy skills due to its narrower focus. Though there is less emphasis on outcomes on parents’ attitudes, practices, and skills as well as other domains of children’s development, studies have sometimes reported potential effects of intervention on these aspects as well. For example, researcher-designed interventions in East Asia have generally reported positive results. In Hong Kong, Chow et al. (2008) tested the effectiveness of a 12-week dialogic reading intervention. Results showed that the program could promote kindergarteners’ improvement in Chinese vocabulary and reading interest, and children whose parents received explicit metalinguistic training also demonstrated improvements in character recognition and morphological awareness (Chow et al., 2008). Cheung and McBride (2017), on the other hand, conducted a 4-week intervention program for kindergarten children who were relatively unskilled in the numeracy domain. Children who completed mathematics workbooks with their parents improved on their addition skills, whereas children who played number board games with their parents demonstrated increased scores in measures of rote counting, numeral identification, and mathematics interest. In a third group wherein parents received additional training on how to play number board games more effectively, the children demonstrated improvements in rote counting, numeral identification, addition, and mathematics interest from pre- to post-test (Cheung and McBride, 2017). Meanwhile, in a case study conducted in Japan, a young girl demonstrated certain improvements in mathematics after being given a simple mathematics quiz game to play at home over a 3-year period (Watanabe, 2019). Although these studies normally focused on children’s cognitive outcomes, researchers have sometimes examined the positive impacts of intervention on parental attitudes and parent-child relationships. In a study involving a 7-week paired reading program for preschoolers and their parents in Hong Kong, the program was observed to not only benefit the preschoolers but also their parents (Lam et al., 2013). Preschoolers demonstrated better word recognition skills, reading fluency, and motivation in reading, whereas parents increased in their self-efficacy in helping children to read after joining the program. Parents also reported an improved relationship with their children.

In South and Southeastern Asia, programs implemented by researchers and non-government organizations were generally found to have positive results, and effects on parents were sometimes reported as well. In India, two types of home reading programs that involved either child-facilitated reading or parent-child reading were both effective in improving children’s English reading skills relative to a control group (Shah-Wundenberg et al., 2013). In another study, the combined effect of an intervention that targeted maternal literacy and encouraged mother-child activities improved literacy and numeracy skills among mothers and children (Banerji et al., 2017). The two programs administered individually were similarly effective for children’s numeracy and mothers’ literacy and numeracy scores, but not for children’s literacy scores. Mothers who participated in the programs also demonstrated stronger beliefs about their responsibility over their children’s education and were more likely to be involved in their children’s homework. In the Philippines, a parent education program with a significant reading and storytelling component (First Read) was found to increase home reading behavior and children’s language and emergent literacy skills (Leer and Teodosio, 2016). In another study, a parent coaching program that focused on dialogic reading, early literacy activities, or early numeracy games resulted in improved children’s skills in the specific domain targeted (Dulay et al., 2019). Finally, a combined family math program in Singapore, which involved both parent workshops and a parent newsletter, resulted in the greatest gains in math scores compared to a workshop-only, newsletter-only, or control condition (Ho, 2007). However, no treatment effects were observed on parental involvement, encouragement, and confidence outcomes.

A positive trend of results for the home learning environment was also found for interventions implemented in Central and Western Asia. In Whitsel and Lapham’s (2014) study, referred to as a parent empowering program, in Tajikistan, parents re-learned mathematics and reading to support children’s learning at school. Results showed that parents, especially young mothers, demonstrated more confidence, self-esteem, and control toward their children’s literacy as a result. Also, all family members were encouraged to get involved in learning activities with children, including counting, painting, singing, and poetry. In the beginning of the program, most of the Tajikistani parents held the opinion that they should not begin any pre-literacy or pre-numeracy skills before school. After participating in the program, parents expressed the belief that early learning is useful for children’s future performance, and that they should focus on their children’s early literacy and numeracy (Whitsel and Lapham, 2014). Unfortunately, no direct evaluation of intervention effects on children’s outcomes was conducted following this program. Meanwhile, in a study by Aram and Levin (2014) with low socioeconomic status families in Israel, children showed significant improvement in linguistic competencies after joining a mediated reading program with their parents. Children’s alphabetic skills improved the most after joining a mediated writing program with their parents, though the mediated reading program was also found to bring positive impacts on children’s alphabetic skills.

In general, broad parent education programs and focused literacy or numeracy interventions both have the potential to positively impact the home learning environment, and ultimately, children’s literacy and numeracy development. Both types of programs are valuable for different reasons. Broad parent education programs acknowledge the multiple overlapping concerns that could keep parents from fostering their children’s literacy and numeracy development, whereas focused programs can provide parents with techniques that work very well on a particular area of concern for children’s learning. In general, more detailed process documentation and more investigations of long-term intervention effects are needed to understand how such programs can be more effective at improving home learning environments and children’s outcomes in Asia.

## Discussion

This review paper has aimed to examine the learning-related beliefs and attitudes of parents in Asia, their home literacy and numeracy practices, the role of the home literacy and numeracy environments in the literacy and numeracy development of young children in Asia, and the effectiveness of programs that aim to improve the home literacy and numeracy environments in Asia. Generally speaking, our review shows that the home learning environments created by parents in Asia are generally consistent with their educational goals and aspirations for their children and are conducive to children’s early literacy and numeracy development. While broad parent education programs have positively benefited children’s outcomes in several instances, focused interventions are more consistent at producing direct (but potentially short-term) impacts on children’s literacy and numeracy skills.

To what extent are the home literacy and numeracy environments in Asia similar or different from those in the West? As mentioned at the beginning of this paper, we cannot give a solid answer to the question given the limited number of relevant cross-cultural studies in the literature. However, the above review does provide some initial insights into the home literacy and numeracy environments in Asia in relation to those in Western societies. Consistent with the situations observed in the West, the home learning environments tend to play a critical role in children’s early development. Generally, the greater the number of home resources available at home and the higher the frequency of home learning activities, the better children’s literacy and numeracy competence and interest. Moreover, parents can often be coached to provide more stimulating home literacy and numeracy environments, which in turn benefits children’s development. Meanwhile, there are at least three issues about the home literacy and numeracy environments in Asia that are not commonly observed or discussed in Western contexts. First, some parents in Asia, especially those in East Asia, tend to place great emphasis on academic achievement and their own responsibility to help children learn at home (Yamamoto and Brinton, 2010; Byun et al., 2012; Bray, 2013). Play is not always favored as it is associated with laziness (Leung, 2011; Luo et al., 2013). Under the influence of the Western idea of child-centeredness, parents’ beliefs appear to be changing, however (Rao and Li, 2009; Fung and Cheng, 2012; Lin and Yawkey, 2013). Second, several studies appear to acknowledge the role of non-parental household members in fostering children’s development in Asian homes, which sometimes span three generations and might even involve non-family members such as domestic helpers (Chung and Koo, 2001; Dulay et al., 2017; Rao et al., 2017; Su et al., 2020). Third, there is a great demand to learn multiple languages in these contexts, as often the children are growing up in multilingual environments (Joshi, 2015; Adamson, 2018; Wang, 2018). In many contexts, children have to be proficient in languages they do not necessarily speak at home in order to attain educational success.

Looking more closely, it should be noted that home environments across different contexts in Asia comprise a certain degree of heterogeneity in parental beliefs, home practices, associations between the home learning environment and child outcomes, and effectiveness of parent training programs. For instance, parents in some places seem to vary in their perceived importance of developing children’s reading and writing competencies prior to formal school entry, as well as the roles of homes and play in children’s learning (Lee, 2002; Howell et al., 2016; Chan, 2012). They also reported different frequencies in various formal and informal home learning activities with children (Kim, 2009; Howell et al., 2016; Huang et al., 2017; Inoue et al., 2018; Cheung et al., 2020). Though these differing parental beliefs and home practices may be attributed to inexplicable variance, individual differences between participants, and different research methodologies adopted across studies, they may also emerge from variations in cultural values and social situations of different places within the vast region of Asia. Specifically, as influenced by Confucianism and the competitive social environment, parents in certain places such as Mainland China, Hong Kong, Japan, Korea, and Singapore, tend to hold higher academic expectations for children and thus invest more in fostering children’s literacy and numeracy skills in the early years (Yamamoto and Brinton, 2010; Byun et al., 2012; Bray, 2013; Luo et al., 2013). On the other hand, Israel is a developed, industrialized country with individualistic values as the dominant culture, though it is also a highly familial society with strong emphasis on communal values (Scharf, 2014). It is thus not surprising that showing warmth toward children, supporting children’s autonomy support and setting expectations for children’s appropriate behaviors are valued more by Israeli parents than providing children with academic-related materials and activities at home (Aram et al., 2020).

Furthermore, our review shows that home learning resources and activities were not uniformly impoverished among the low- and middle-income economies in a given region; in fact, there is considerable variability in home resources and practices even within high-income economies such as Singapore. Home learning resources and activities also demonstrate non-uniform relationships with children’s skills across home learning variables, literacy and numeracy sub-domains, family members, and sample characteristics. One possible source of such disparities is that parents’ education and income levels, as well as other demographic variables (e.g., family size) and personal variables (e.g., personal abilities, feelings, and experiences with literacy and numeracy), may affect their parental beliefs about early literacy and numeracy learning, the extent to which they can enact such beliefs, and the effectiveness of their home practices in promoting various aspects of child development (Wolff and Breit, 2012; Chow et al., 2017; Dulay et al., 2019; Cheung et al., 2020). Further examination is thus warranted to enhance our understanding of how various personal, socioeconomic, and cultural factors interact with each other to contribute to the diverse home learning environments within and across regions in Asia.

### Limitations

There are several limitations to note in this review. First, though we endeavored to include studies from all possible territories in Asia, we did not find studies from many important and representative locations, including Afghanistan, Azerbaijan, Armenia, Brunei Darussalam, Kuwait, Malaysia, and the Maldives, as well as many others that fit the scope of the review. One possible reason for this omission is that the studies conducted in these places, if any, are not written in English or are not easily accessible on the internet. Second, for some locations (especially those in Southern, Southeastern, Central, and Western Asia), the best-known studies have been funded through initiatives by governmental (e.g., USAID) or non-government organizations (e.g., Save the Children and UNICEF) and are reported in the gray literature. In contrast, many studies from East Asia are peer-reviewed articles and have been funded largely by academic grants. Hence, the research frameworks used, nature of sample recruitment, and degree of detail in reporting tended to vary across sources. Third, the samples in many of these studies are not representative of the whole population. Therefore, it is not advisable to use the results of a single study to make sweeping generalizations about the situation of a particular country or territory; comparison of the situation across contexts should be done with great caution. Meanwhile, it is not surprising to see that there are significant variations across contexts in Asia, as well as across samples within a context. Indeed, apart from the large list of countries and territories covered, the generalizability of any single study is limited by the diversity of socioeconomic and linguistic profiles among people living in these regions. Fourth, there are relatively fewer studies on the home numeracy environment than on the home literacy environment. Studies on the quality of the home learning environment, such as the content and style of parent-child interactions during home learning activities, are also limited. Last but not least, there is a great variety of terms used in the literature to describe the home learning environment. This lack of standardization in the terminology used as well as the assessment frameworks made the search for relevant articles and drawing comparisons across studies more difficult.

### Future Directions

As shown above, there are several topics regarding the home literacy and numeracy environments in Asia that are still under-explored and require further investigation. Indeed, there is a great need for more research on this diverse and huge population of those from Asia, given that children’s immediate learning environments (including the home learning environments) are affected by the larger sociocultural contexts in which they live (Bronfenbrenner, 1979); at the same time, children and families play an active role in sustaining or changing the cultural practices within the group (Miller and Goodnow, 1995). We briefly highlight just four suggested research directions here. First, more cross-cultural studies with comparable samples can be conducted. For example, we may wish to explore relatively wealthier and poorer citizens across countries in order to understand the interplay of culture and relative income level for educational attainment. Second, more longitudinal studies should be carried out to investigate the direct and indirect effects of the home learning environments on children’s literacy and numeracy development. We may also compare the relative role of the home and school experience over time. Third, the mechanisms underlying the interactions between parental beliefs and attitudes, parental practices, and children’s outcomes can be explored further. Specifically, we may evaluate whether there are any gaps between parents’ beliefs and practices and identify which types of parents are more likely to have such a gap. Finally, more work needs to be done in order to identify underlying sources of variability in the home learning environments across countries and to find the best routes by which to empower homes that fit various Asian contexts. In particular, the optimal content, form and intensity of programs for parents in different sociocultural settings can be examined.

### Conclusion

This review paper is one of only a few, if any, to examine the home literacy and numeracy environments across different regions in Asia. On the one hand, we have discussed how the home learning environments in Asia are shaped by some sociocultural variables. On the other hand, by appreciating the sheer diversity in home learning environments and children’s experiences in the contexts that we have covered, we have been able to identify some features of the home environment that warrant further exploration, such as the underlying role of cultural values and social situations in determining how parents provide educational experiences to their children at home, the relatively underexplored role of non-parental caregivers in shaping the home environment, the need to identify context-relevant mediators and moderators that underlie the relationship between the home learning environment and children’s outcomes, and the need to identify the most effective means for delivering intervention in these skill domains. Overall, this review paper enhances our understanding of the role of sociocultural factors in shaping home environments, and thus children’s early development, in Asia. Beyond that, we have identified potential avenues where we can deepen our understanding of how homes can support children’s literacy and numeracy around the world.

## Author Contributions

SC, KD, and CM developed the research idea. SC, KD, XY, FM, and CM wrote and reviewed the manuscript. All authors contributed to the article and approved the submitted version.

### Conflict of Interest

The authors declare that the research was conducted in the absence of any commercial or financial relationships that could be construed as a potential conflict of interest.
